# Supplementary material for: Complementary mesoscale dynamics of spectrin and acto-myosin shape membrane territories during mechanoresponse
Source: Nat Commun. 2020 Oct 9;11:5108. doi: 10.1038/s41467-020-18825-7 (PMC7547731; doi:10.1038/s41467-020-18825-7)
Supplement: Supplementary file 1 — Supplementary Information [file 41467_2020_18825_MOESM1_ESM.pdf]

Complementary Mesoscale Dynamics of Spectrin and Acto-Myosin shape Membrane Territories during Mechanoresponse

Ghisleni A., et al.

Supplementary Information

-Supplementary Figures

-Supplementary Movies

-Supplementary Tables

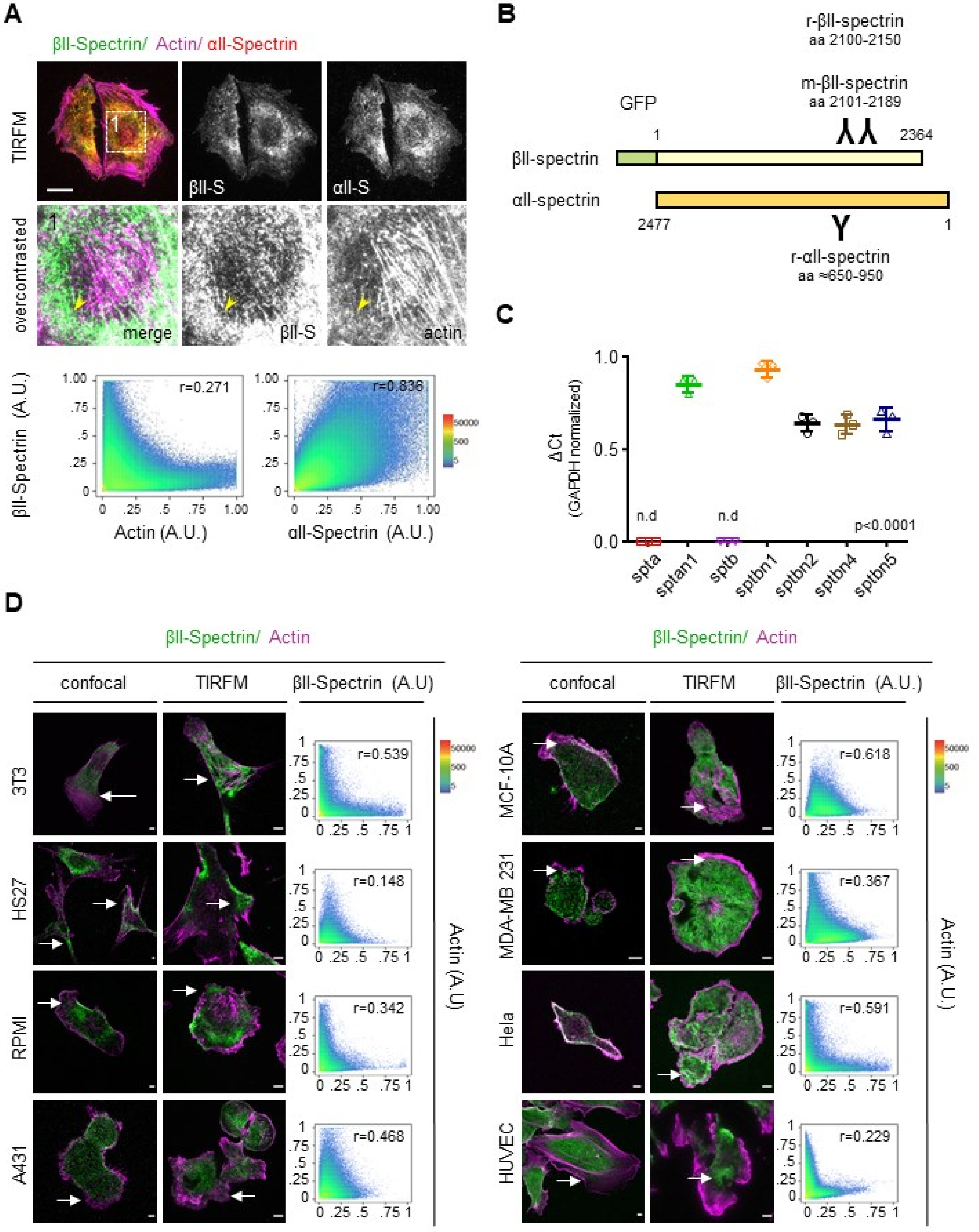
A
βII-Spectrin/ Actin/ αII-Spectrin
TIRFM
βII-S
αII-S
overcontrasted
merge
actin
r=0.271
r=0.836
βII-Spectrin (A.U.)
Actin (A.U.)
αII-Spectrin (A.U.)
B
r-βII-spectrin
aa 2100-2150
m-βII-spectrin
aa 2101-2189
GFP
1
2364
βII-spectrin
αII-spectrin
2477
r-αII-spectrin
aa ≈650-950
C
ΔCt
(GAPDH normalized)
n.d
p<0.0001
spta
sptan1
sptb
sptbn1
sptbn2
sptbn4
sptbn5
D
βII-Spectrin/ Actin
confocal
TIRFM
βII-Spectrin (A.U)
3T3
HS27
RPMI
A431
r=0.539
r=0.148
r=0.342
r=0.468
Actin (A.U)
βII-Spectrin (A.U.)
MCF-10A
MDA-MB 231
Hela
HUVEC
r=0.618
r=0.367
r=0.591
r=0.229
Actin (A.U.)

Supplementary Figure 1. βII-Spectrin and Actin complementarity is observed in multiple cell lines.

A) Representative images of MEFs immunostained for βII-spectrin (green), αII-spectrin (red) and F-actin (magenta), visualized by TIRFM (scale bar: 10 µm). Overcontrasted magnification of βII-spectrin (green) and F-actin (magenta) corresponding to 1 (white dashed box) are presented. βII-spectrin is excluded from stress fibers, only low-abundance cortical actin is visible in the spectrin-rich zone (yellow arrowhead). Correlation analysis of pixel intensities between the different fluorescent channels are reported in the scatter plots, related Pearson's correlation coefficients (r) are reported. B) Schematic representation of the α/β-spectrin dimer and the epitopes recognized by the spectrin-reacting antibodies implemented in this study (m: mouse-raised monoclonal antibody, r: rabbit-raised polyclonal antibody). C) Gene expression analysis of different spectrin isoforms in MEFs used for this study, measured by qPCR (n=3, n.d.=not detected, data are presented as mean values ± SD, Statistical analysis one-way ANOVA, $p<0.0001$). The genes *SPTAN1* and *SPTBN1* encodes for αII and βII spectrin respectively. D) Different cell lines immunostained for F-actin (magenta) and endogenous βII-spectrin (green). Confocal and TIRFM images are presented, arrows indicate peculiar complementary zones (scale bar: 10 µm). Cross-correlation analysis by scatter plot between the two channels and related Pearson's correlation coefficients are reported for TIRFM images. All images are representative of many cells immunolabelled in n=2 or more independent experiments.

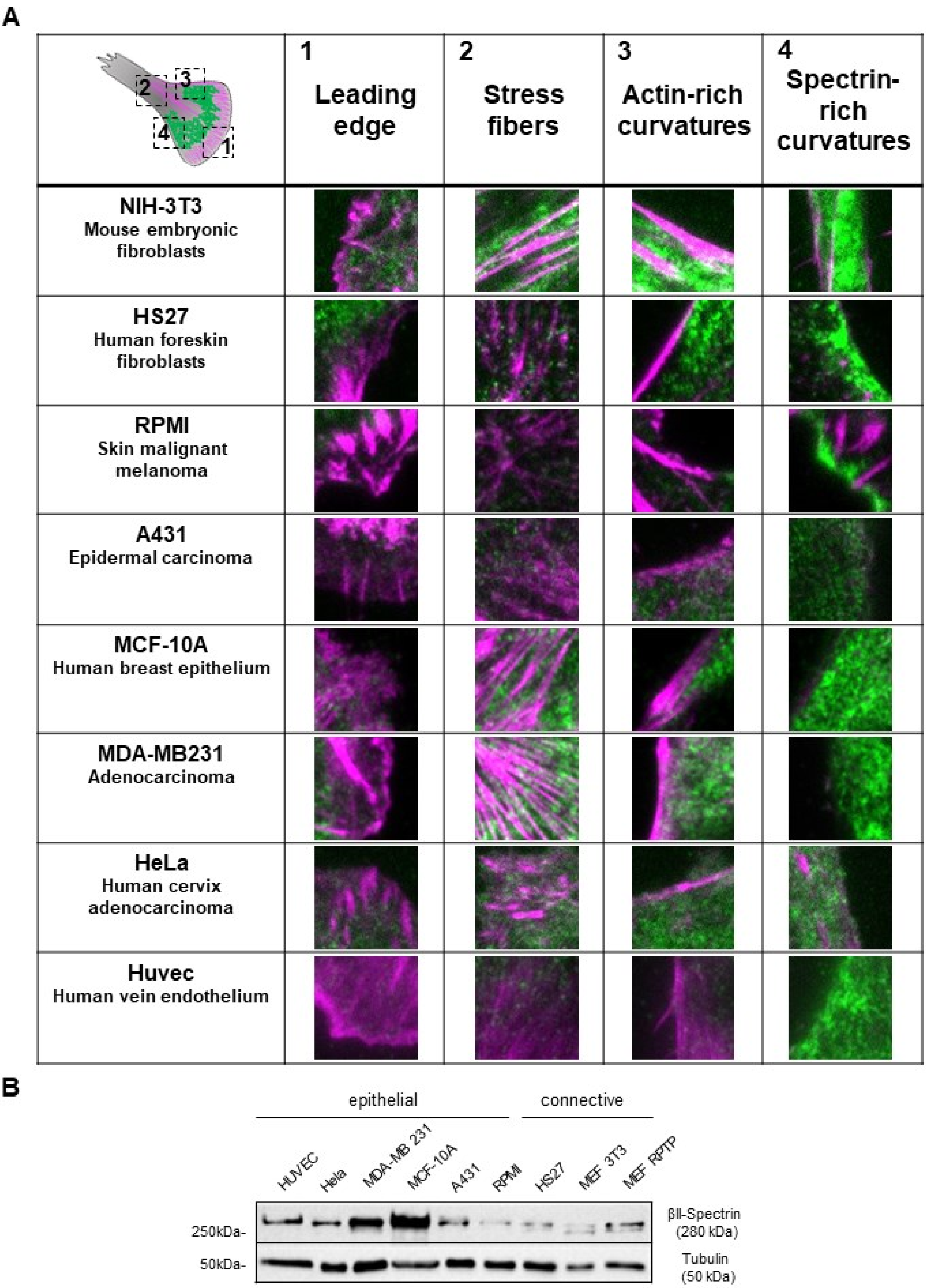
A
1
Leading edge
2
Stress fibers
3
Actin-rich curvatures
4
Spectrin-rich curvatures
NIH-3T3
Mouse embryonic fibroblasts
HS27
Human foreskin fibroblasts
RPMI
Skin malignant melanoma
A431
Epidermal carcinoma
MCF-10A
Human breast epithelium
MDA-MB231
Adenocarcinoma
HeLa
Human cervix adenocarcinoma
Huvec
Human vein endothelium
B
epithelial
connective
HUVEC
Hela
MDA-MB 231
MCF-10A
A431
RPMI
HS27
MEF 3T3
MEF RPTP
250kDa-
50kDa-
βII-Spectrin (280 kDa)
Tubulin (50 kDa)

Supplementary Figure 2. Zooms of βII-Spectrin and Actin complementary territories

A) TIRFM images (2x2 µm) of the different cell lines immunostained for endogenous βII-spectrin (green) and F-actin (magenta). Cell zones (as listed in Figure 1 A for MEFs) highlighting βII-spectrin/actin complementarity are reported. B) βII-spectrin protein expression in different cell lines analyzed by western blot in total cell lysates. All images are representative of many cells immunolabelled in n=2 or more independent experiments.

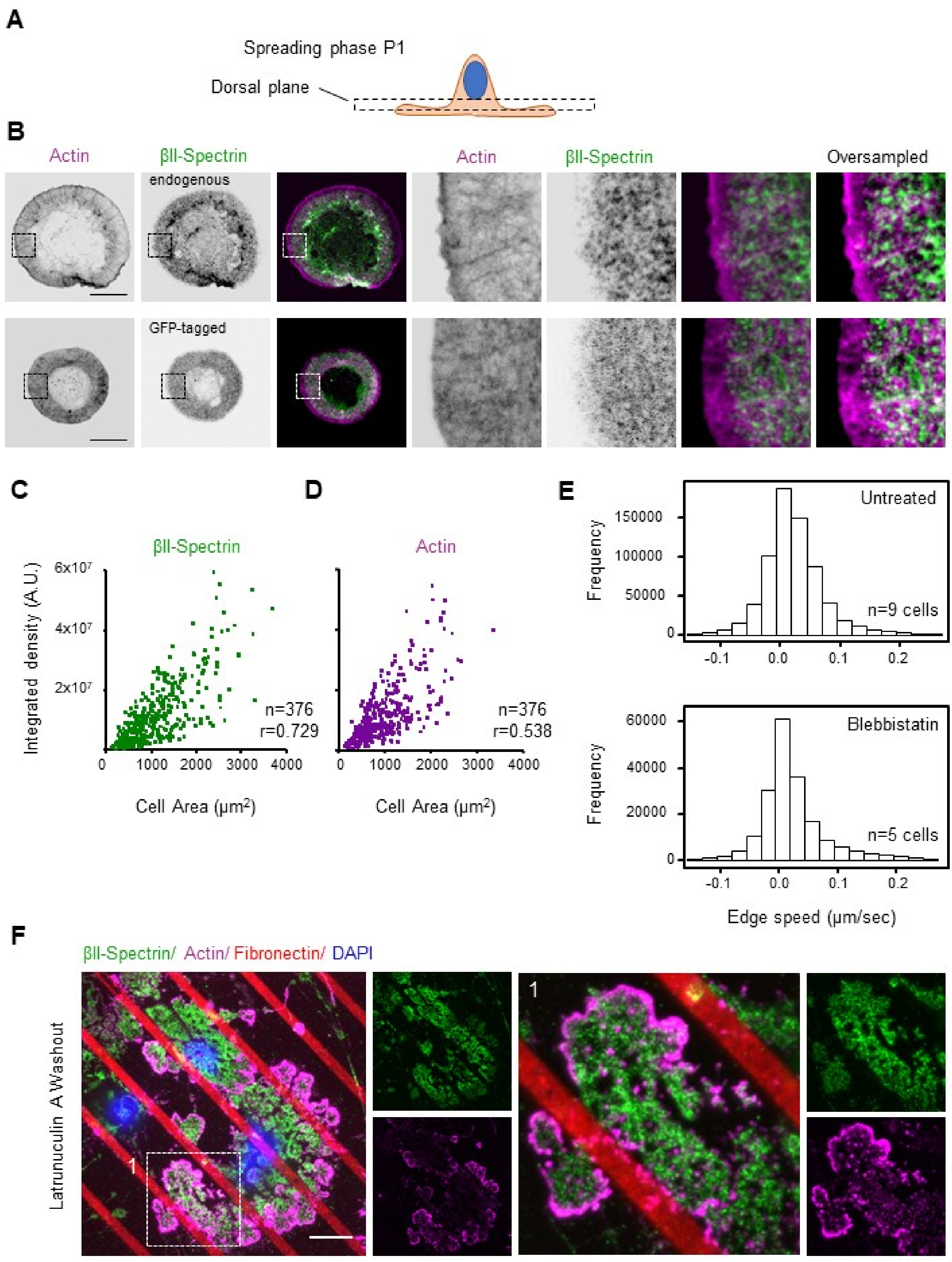
A
Spreading phase P1
Dorsal plane
B
Actin
βII-Spectrin
endogenous
GFP-tagged
Actin
βII-Spectrin
Oversampled
C
βII-Spectrin
Integrated density (A.U.)
n=376
r=0.729
Cell Area (µm²)
D
Actin
n=376
r=0.538
Cell Area (µm²)
E
Untreated
Frequency
n=9 cells
Blebbistatin
n=5 cells
Edge speed (µm/sec)
F
βII-Spectrin/ Actin/ Fibronectin/ DAPI
Latrunuculin A Washout
1

Supplementary Figure 3. βII-Spectrin organization in P1; βII-Spectrin/area during spreading; βII-Spectrin and Actin recovery after Latrunculin A washout in cortex-mimicry zones

A-B) MEFs fixed during P1, immunolabelled for endogenous F-actin (magenta) and βII-spectrin (green, endogenous and GFP-tagged), and analyzed by 3D confocal microscopy. Optical sectioning is optimized to resolve the cortex on the cell dorsal plane during P1, as shown in the cartoon (scale bar: 20 μm). C-D) Cells fixed at different time points after seeding (between 5-20 minutes) and immunolabelled for endogenous βII-spectrin and F-actin. Projected cell area and fluorescence integrated intensities in TIRFM for the two proteins are reported, displaying linear correlation (n=376 cells, see Table 1). E-F) Total data point distribution of the graphs shown in Figure 2E and 2G, outliers were excluded from the analysis (threshold 0.0007; untreated: n=9 cells; blebbistatin: n=5 cells). Both analysis showed normal gaussian distribution between the physiological speed range of -0.1 μm sec^{-1} and +200 μm sec^{-1}. F) MEFs seeded on micropatterned fibronectin-coated lines. TIRFM images of cells fixed during the washout phase after latrunculin A treatment are shown (green: βII-spectrin, magenta: F-actin, red: fibronectin. Scale bar: 20 μm). DAPI (blue) is visualized in EPI mode to discriminate intact cells from debris. The white dashed box (1) is zoomed to highlight peculiar actin nodes formation in non-adhesive cell cortex. All images are representative of many cells immunolabelled in n=2 or more independent experiments.

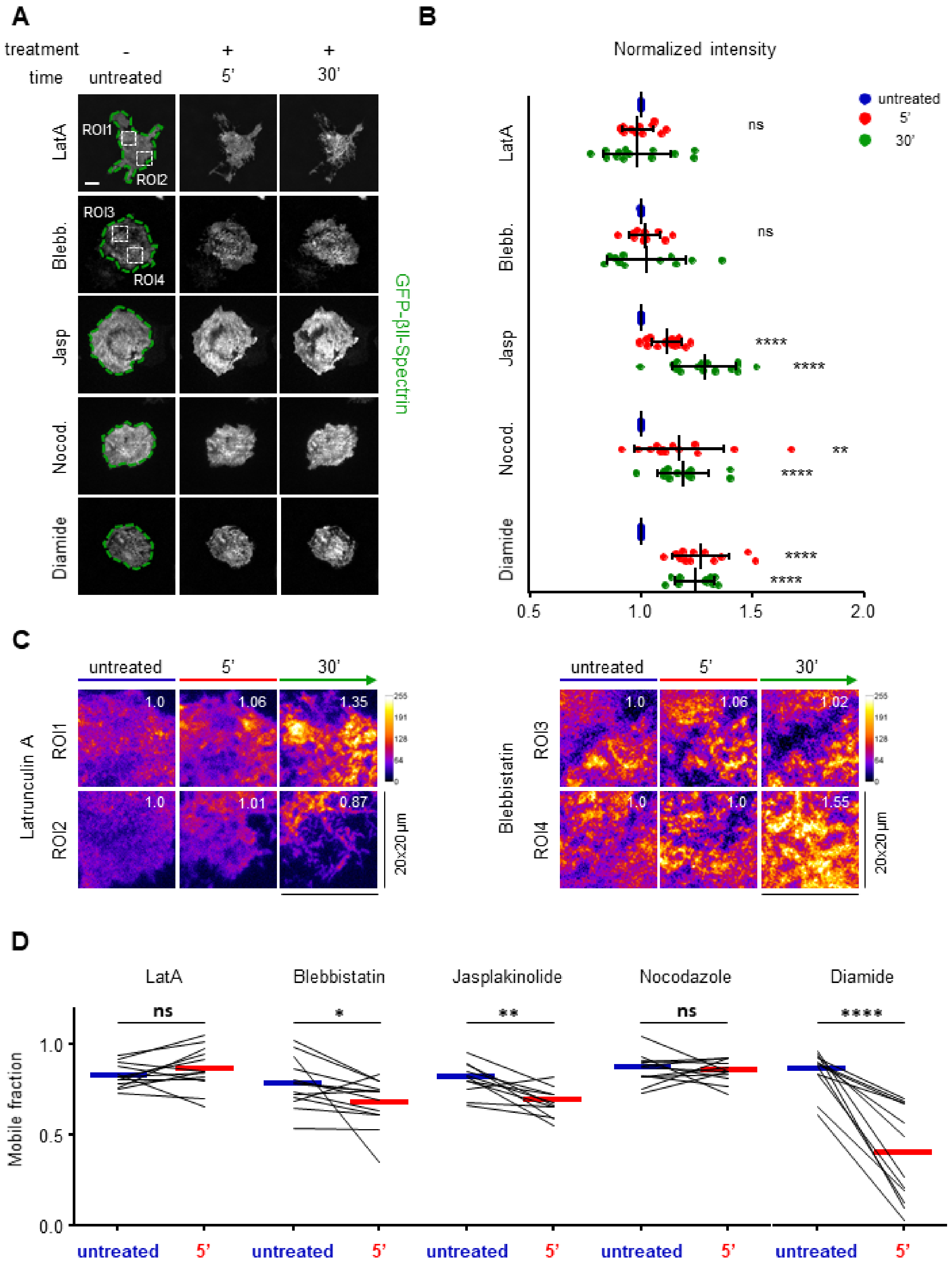
A
treatment - + +
time untreated 5' 30'
LatA
Blebb.
Jasp
Nocod.
Diamide
ROI1
ROI2
ROI3
ROI4
GFP-βII-Spectrin
B
Normalized intensity
untreated
5'
30'
ns
ns
****
****
**
****
****
****
0.5
1.0
1.5
2.0
C
untreated 5' 30'
Latrunculin A
ROI1
ROI2
1.0 1.06 1.35
1.0 1.01 0.87
20x20 µm
Blebbistatin
ROI3
ROI4
1.0 1.06 1.02
1.0 1.0 1.55
D
LatA
Blebbistatin
Jasplakinolide
Nocodazole
Diamide
ns
*
**
ns
****
Mobile fraction
1.0
0.5
0.0
untreated 5'
untreated 5'
untreated 5'
untreated 5'
untreated 5'

Supplementary Figure 4. βII-Spectrin perturbation by cytoskeletal impairing drugs

A) GFP-βII-spectrin expressing MEFs imaged by live TIRFM during administration of cytoskeletal impairing drugs are shown before (-) and during (+) the treatments (scale bar: 20 µm). Whole-cell mean fluorescence intensities are normalized to the pre-treatment frames (blue circles), and plotted in B at 5 minutes (red circles) and 30 minutes (green circles) of treatment (n=12 cells, data are presented as mean values ± SD, one-way Anova with multiple comparisons: ****p<0.0001, **p=0.0095). C) Four ROIs (20x20 µm) related to drug treatments with no apparent fluctuations in GFP-βII-spectrin intensities are presented (ROIs related to cells in panel A treated with latrunculin A and blebbistatin). This ROIs showed differential GFP-βII-spectrin reaction depending on different location. The normalized intensities across the entire projected cell area underestimate the mesoscale differential behavior of the meshwork, mean intensities normalized to the untreated frame are reported on the top-left corner of each image. D) FRAP single cell analysis of mobile fractions: the same graph presented in Figure 4 F is reported here highlighting the difference in mobile fraction within the same cell (connected by black lines) before (blue) and 5' after treatment (red) (Statistical analysis: paired Student's t-test two-tailed, data are presented as mean values ± SD, * p=0.0413, ** p=0.0021, **** p<0.0001, n=12 cells).

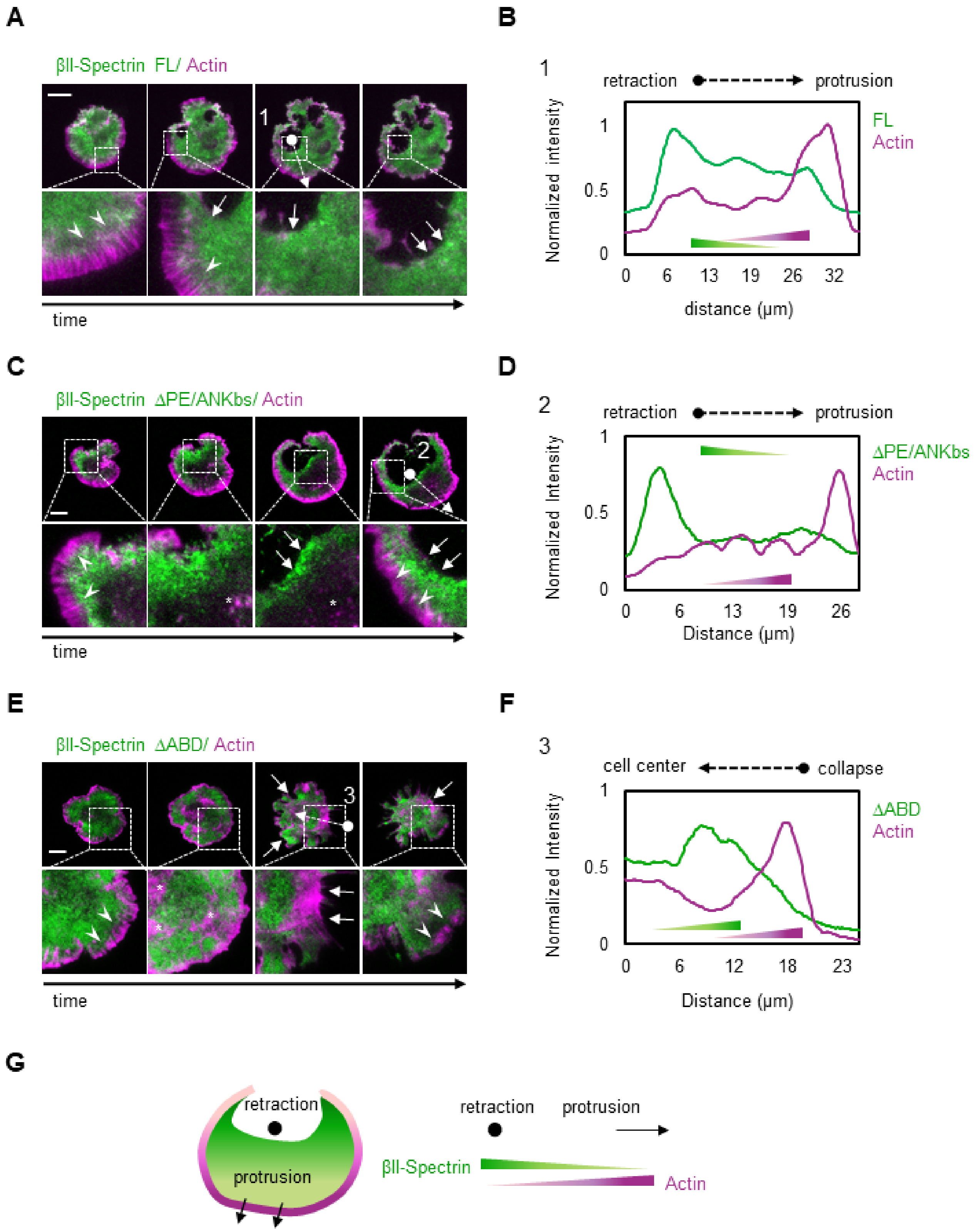
A
βII-Spectrin FL/ Actin
1
time
B
1
retraction protrusion
Normalized intensity
1
0.5
0
0 6 13 19 26 32
distance (µm)
FL
Actin
C
βII-Spectrin ΔPE/ANKbs/ Actin
2
time
D
2
retraction protrusion
Normalized Intensity
1
0.5
0
0 6 13 19 26
Distance (µm)
ΔPE/ANKbs
Actin
E
βII-Spectrin ΔABD/ Actin
3
time
F
3
cell center collapse
Normalized Intensity
1
0.5
0
0 6 12 18 23
Distance (µm)
ΔABD
Actin
G
retraction
protrusion
retraction protrusion
βII-Spectrin
Actin

Supplementary Figure 5. βII-Spectrin FL, ΔPE/ANKbs and ΔABD accumulate at negative curvatures that spontaneously form during cell spreading/polarization

A-C-E) Representative images of spontaneous retractile events observed in MEFs expressing GFP-βII-spectrin variants during cell spreading by live TIRFM (GFP-βII-spectrin variants in green and RFP-Actin in magenta, scale bar: 20 µm). Relevant events are highlighted by the dashed boxes and zoomed in the lower panels: protruding zones are indicated by white arrowheads, retracting zones by white arrows. Line scan analysis of arrows with circular ends (1-2-3) are reported in B-D-F for both proteins, directionality is reported above the graphs. G) Cartoon model of Actin/βII-spectrin opposite polarity exploited by protrusion/retraction events during the polarization phase of cell spreading. Images are representative of many cells in n=3 or more independent experiments.

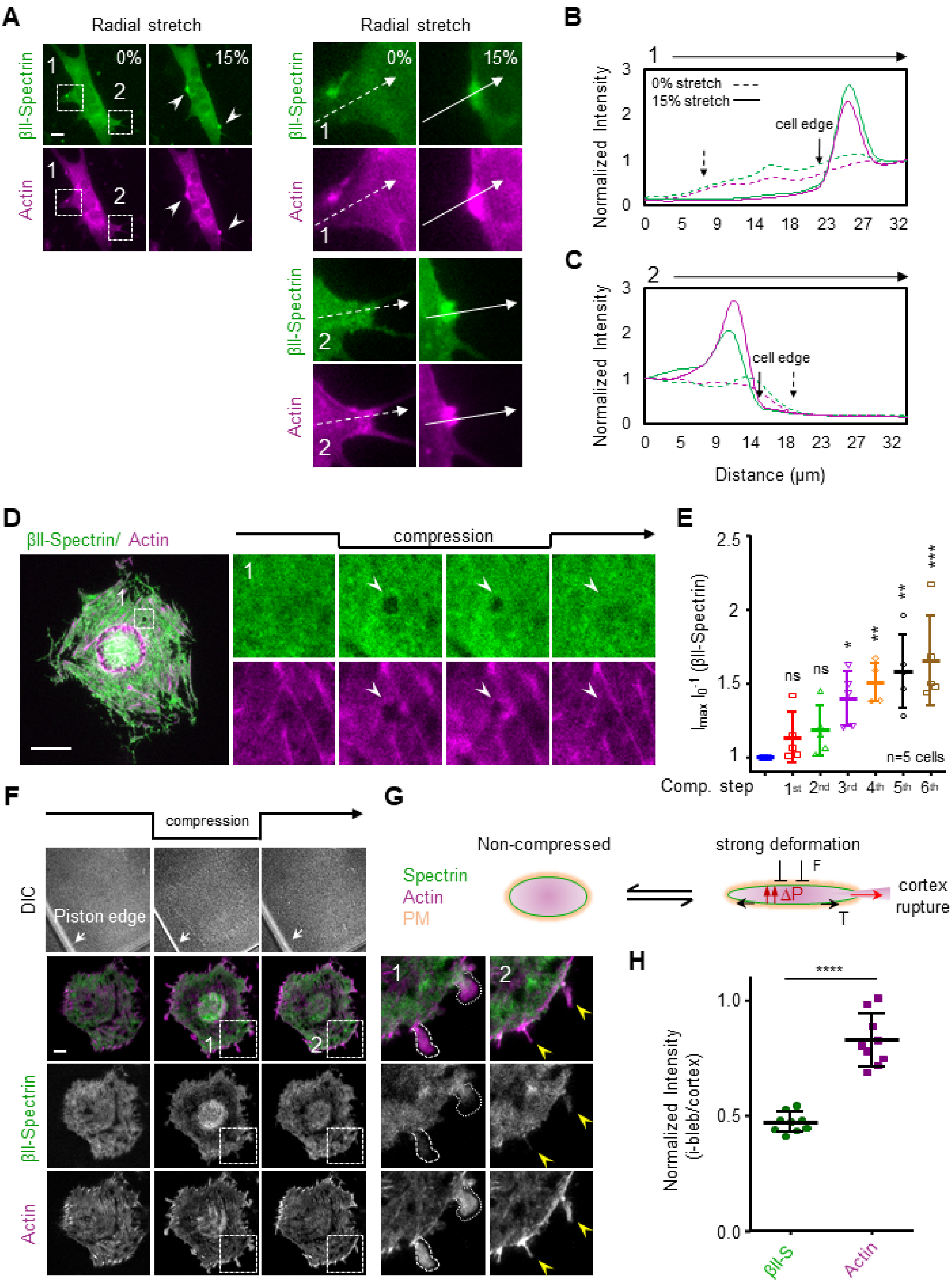

A
Radial stretch
βII-Spectrin
Actin
0%
15%
1
2
B
C
0% stretch
15% stretch
cell edge
Normalized Intensity
Distance (μm)
D
βII-Spectrin/ Actin
compression
E
$I_{max}\ I_0^{-1}$ (βII-Spectrin)
ns
n=5 cells
Comp. step
1st 2nd 3rd 4th 5th 6th
F
DIC
Piston edge
G
Non-compressed
strong deformation
Spectrin
Actin
PM
F
ΔP
T
cortex rupture
H
Normalized Intensity (i-bleb/cortex)
βII-S
Actin

Supplementary Figure 6. βII-Spectrin reactions to mechanical perturbations highlight the interplay with Actin also in collapsed protrusions, vesicles and blebs

A) Bi-axial cell stretching experiment of GFP-βII-spectrin (green) and RFP-actin (magenta) expressing MEFs, imaged by live EPI fluorescence microscopy (scale bar: 10 µm). Dashed boxes 1 and 2 highlight two specific cell protrusions that detached upon 15% stretch. Intensity profile across these processes (white dashed (0%) and full (15%) arrow) are plotted in B and C. Representative images of n=16 cells in 3 independent experiments. D) The same cell presented in figure 6 E is reported (scale bar: 20 µm). 1) Panel of images corresponding to the white dashed box 1, highlighting the appearance of a vesicular structure (white arrowhead) upon compression, devoid of both βII-spectrin and actin, resembling the clearance effect observed underneath the nucleus. E) Quantification of GFP-βII-spectrin maximal intensity at the cell body during the sequential compression protocol, normalized to the pre-compression phase (Statistical analysis one-way Anova with multiple comparison, * $p=0.0153$, ** $p<0.0015$, *** $p<0.0001$, data are presented as mean values ± SD, n=5 cells). F) Maximal compression experiments of GFP-βII-spectrin (green) and RFP-actin (magenta) expressing MEFs: compression strain is gradually increased until bleb formation is induced (scale bar: 10 µm). Cortex rupture mechanism is schematized in G: key elements are the variation in intracellular pressure (ΔP) and cortex tension (T) during compression. Representative images of n=4 independent experiments are shown during (1) and upon release of compression (2), when induced blebs are resorbed into tubular-like actin enriched structures (yellow arrowheads). Actin and βII-spectrin content in the blebs compared to the adjacent cell body are quantified in H (Statistical analysis: unpaired Student's t-test two-tailed, * $p=0.0000338$, data are presented as mean values ± SD, n=9 cells in 4 independent experiments).

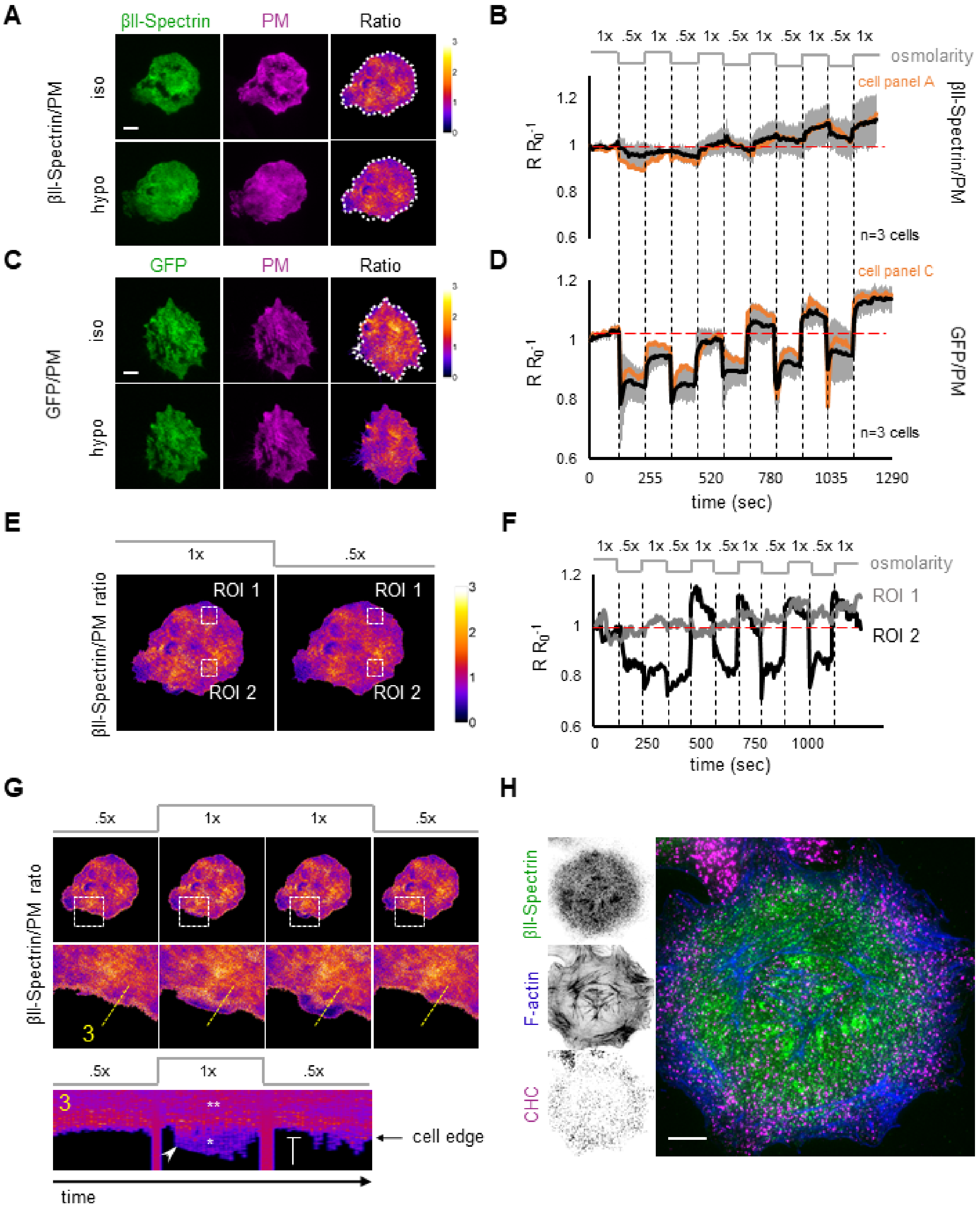
A
βII-Spectrin
PM
Ratio
βII-Spectrin/PM
iso
hypo
B
1x .5x 1x .5x 1x .5x 1x .5x 1x .5x 1x
osmolarity
cell panel A
βII-Spectrin/PM
R R0-1
n=3 cells
C
GFP
PM
Ratio
GFP/PM
iso
hypo
D
cell panel C
GFP/PM
n=3 cells
time (sec)
E
1x
.5x
ROI 1
ROI 2
βII-Spectrin/PM ratio
F
1x .5x 1x .5x 1x .5x 1x .5x 1x .5x 1x
osmolarity
ROI 1
ROI 2
R R0-1
time (sec)
G
.5x
1x
1x
.5x
βII-Spectrin/PM rato
3
cell edge
time
H
βII-Spectrin
F-actin
CHC

Supplementary Figure 7. βII-Spectrin reactions to osmotic changes: global versus local behavior

A) Representative images of GFP-βII-spectrin (green) and PM-marker (magenta) transfected MEFs observed by live TIRFM during osmotic shocks. Five isotonic (1x)-to-hypotonic (0.5x) cycles were applied (B). Initial fluorescent signals were normalized to obtain the non-stoichiometric ratio βII-spectrin/PM (LUT fire, scale bar: 10 µm). Average ratio is plotted in B (black line, n=3 cells, mean ± SD), while the ratio of the cell in A is shown by the orange line. C-D) As positive control, the same protocol was applied to MEFs transfected with soluble GFP (green) and PM-marker (magenta), while GFP/PM ratio (LUT fire, scale bar: 10 µm) is plotted in D (n=3 cells, data are presented as mean values ± SD). Zonal ratio analysis at two extreme cases is reported in E: ROI1 presented high βII-spectrin/PM ratio, while ROI2 displayed lower ratio. As show in the graph (F), the two ROIs behave differently: while ROI1 reacted similarly to the whole-cell analysis presented in B, ROI2 showed an initial decrease of the ratio sustained during the first two iso-to-hypotonic cycles, followed by a compensatory effect that restored the initial ratio during the last four cycles. A similar effect in a different PM zone is presented in G: lamellipodia (dashed box and zoomed in the bottom panel) characterized by high actin and low βII-spectrin content were blocked during hypotonic shocks. Kymograph generated across the dashed yellow line (3): βII-spectrin/PM ratio was low in lamellipodia (* asterisk) compared to the adjacent cell body (** asterisks), and lamellipodia blockage during the hypotonic shock was observed. H) The same cell presented in Figure 7 A is shown: endogenous βII-spectrin (green), clathrin heavy chain (CHC, magenta) and F-actin (blue), imaged by TIRFM (scale bar: 10 µm, images are representative of many cells immunolabelled in n=2 or more independent experiments).

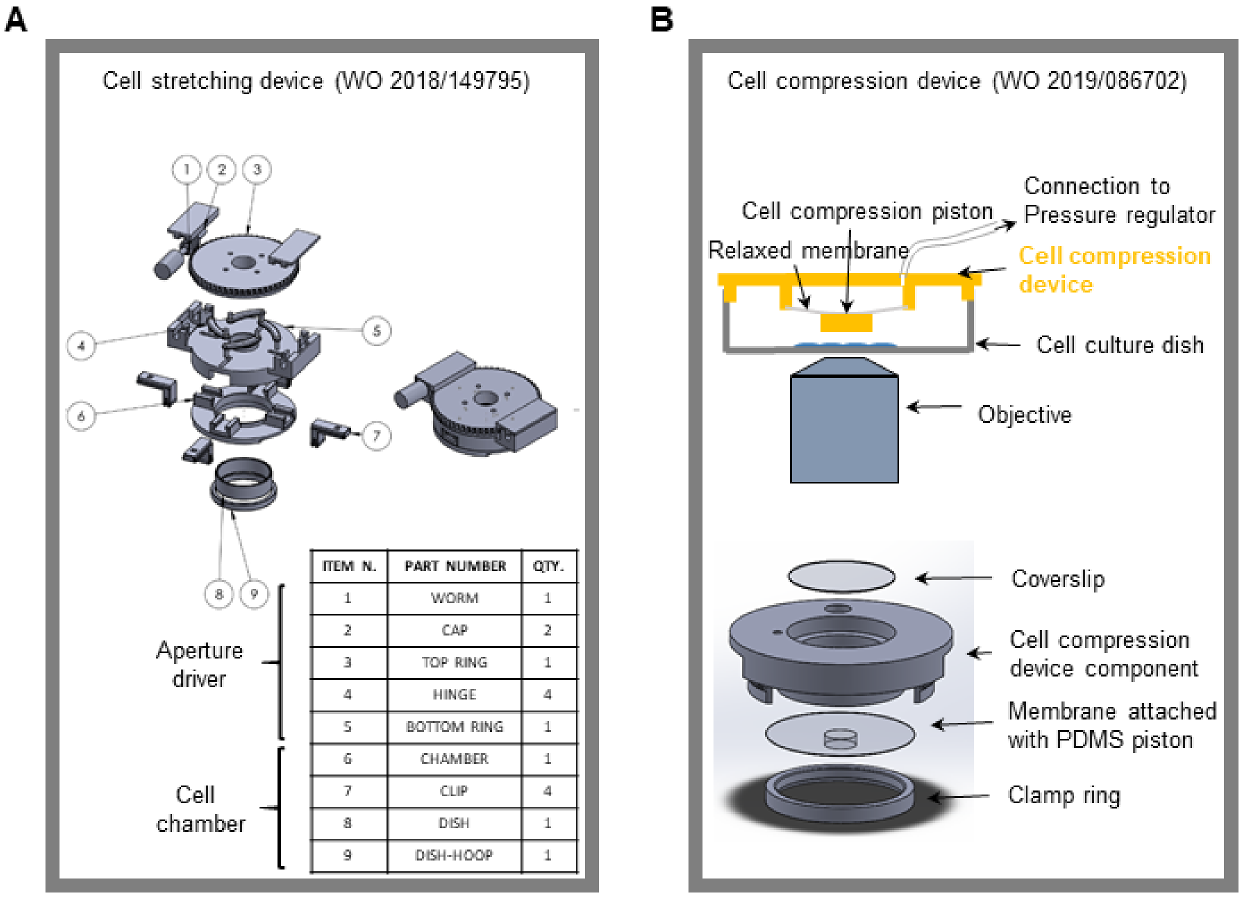

ITEM N.	PART NUMBER	QTY.
1	WORM	1
2	CAP	2
3	TOP RING	1
4	HINGE	4
5	BOTTOM RING	1
6	CHAMBER	1
7	CLIP	4
8	DISH	1
9	DISH-HOOP	1

Supplementary Figure 8. Schemes of the patented devices implemented in this study

Exploded-view drawings of the cell stretching dish (A) and the cell compression device (B), where the main components and assembly order are highlighted.

Supplementary Movies

Supplementary Movie 1 Fibroblast spreading assay: cell edge analysis of GFP-βII-spectrin and RFP-Actin

Supplementary Movie 2 Fibroblast spreading on microprinted fibronectin-coated lines (GFP-βII-spectrin and RFP-Actin)

Supplementary Movie 3 Fibroblast spreading: PIV analysis of GFP-βII-spectrin and RFP-Actin flows

Supplementary Movie 4 Actin and βII-spectrin dynamics during Latrunculin A and Blebbistatin washout experiments

Supplementary Movie 5 Differential βII-spectrin deletion mutants' behavior during spreading

Supplementary Movie 6 βII-spectrin-ΔABD displays edge instability during spreading

Supplementary Movie 7 Cell compression assay

Supplementary Movie 8 Mesoscale dynamics of GFP-βII-spectrin and mCherry-AP2 during osmotic shocks

Supplementary Tables

Supplementary Table 1 Spreading area/spectrin and area/actin linear regression analysis

	Actin Integrated density	Spectrin integrated density
Pearson's correlation coefficient	0.5386254	0.7296664
Linear regression		
Best-fit values		
B0	4.758e+02	4.708e+02
B1	4.459e-05	4.405e-05
Std. Error		
B0	4.260e+01	2.821e+01
B1	3.962e-06	2.345e-06
T value		
B0	11.17	16.69
B1	11.26	18.79
Goodness of Fit		
Degrees of Freedom	371	301
Multiple R-squared	0.2901	0.5324
Adjusted R-squared	0.2878	0.5309
p-value	2.2e-16	2.2e-16
Beta estimate	4.45906e-05	4.40486e-05
Standard error	3.96159e-06	2.344543-06
T value	11.25574	18.78771
P value	7.35929e-25	4.67908e-53
F statistic	7.16465e-25	7.16465e-25
F	4.45906e-05	4.40486e-05
Model p	3.96159e-06	2.34454e-06
Outliers	12	12

Supplementary Table 2 Dual FRAP Analysis

	Diamide		Nocodazole		Latrunculin A		Blebbistatin		Jasplakinolide	
	Control	Treated (5′)	Control	Treated (5′)	Control	Treated (5′)	Control	Treated (5′)	Control	Treated (5′)
One-phase association Best-fit values										
Y0	0.009453	0.01164	-0.05037	0.02238	-0.01107	-0.08307	-0.004542	0.002546	0.03635	-0.09776
Plateau	0.8569	0.3446	0.8513	0.8262	0.8294	0.8526	0.8371	0.6676	0.8595	0.6730
K	0.01803	0.01437	0.0196	0.01504	0.01989	0.02544	0.02008	0.01698	0.02168	0.03107
Tau	55.45	69.59	51.01	66.48	50.29	39.3	49.79	58.91	46.13	32.18
Half-time (sec)	38.44	48.24	35.36	46.08	34.86	27.24	34.51	40.83	31.98	22.31
Span	0.8474	0.3329	0.9017	0.8038	0.8405	0.9357	0.8417	0.6701	0.8232	0.7707
Std. Error										
Y0	0.02527	0.03849	0.01835	0.02037	0.02294	0.03224	0.02625	0.02802	0.03465	0.03899
Plateau	0.006448	0.01448	0.004094	0.007067	0.005005	0.004843	0.00564	0.007901	0.006615	0.004377
K	0.000843	0.003078	0.0005928	0.0006812	0.0007993	0.001125	0.0009171	0.00116	0.001277	0.001827
Span	0.02315	0.0336	0.01704	0.01798	0.02135	0.03085	0.02447	0.02539	0.03262	0.03784
95% Confidence Intervals										
Y0	-0.04013 to 0.05903	-0.06389 to 0.08716	-0.08638 to -0.01436	-0.01760 to 0.06236	-0.05608 to 0.03393	-0.1463 to -0.01982	-0.05606 to 0.04698	-0.05753 to 0.05244	-0.03165 to 0.1043	-0.1743 to -0.02125
Plateau	0.8442 to 0.8695	0.3161 to 0.3730	0.8433 to 0.8594	0.8123 to 0.8401	0.8196 to 0.8392	0.8431 to 0.8621	0.8260 to 0.8482	0.6521 to 0.6831	0.8465 to 0.8725	0.6644 to 0.6816
K	0.01638 to 0.01969	0.008330 to 0.02041	0.01844 to 0.02077	0.01371 to 0.01638	0.01832 to 0.02145	0.02324 to 0.02765	0.01828 to 0.02188	0.01470 to 0.01925	0.01917 to 0.02418	0.02749 to 0.03465
Tau	50.80 to 61.05	49.00 to 120.0	48.15 to 54.22	61.05 to 72.96	46.61 to 54.59	36.17 to 43.04	45.70 to 54.69	51.95 to 68.03	41.35 to 52.16	28.86 to 36.38
Half-time	35.21 to 42.32	33.96 to 83.21	33.38 to 37.59	42.32 to 50.57	32.31 to 37.84	25.07 to 29.83	31.67 to 37.91	36.01 to 47.15	28.66 to 36.15	20.00 to 25.22
Span	0.8020 to 0.8928	0.2670 to 0.3988	0.8683 to 0.9351	0.7685 to 0.8391	0.7986 to 0.8824	0.8751 to 0.9962	0.7936 to 0.8897	0.6203 to 0.7199	0.7592 to 0.8872	0.6965 to 0.8450
Goodness of Fit										
Degrees of Freedom	1149	1149	1053	1053	1149	1149	957	1149	1149	1149
R square	0.6867	0.1122	0.8441	0.7471	0.7357	0.6725	0.7202	0.5177	0.5529	0.5236
Absolute Sum of Squares	15.39	47.97	6.027	10.69	10.97	14.27	9.822	20.59	21.82	14.05
Sy.x	0.1157	0.2043	0.07566	0.1007	0.09771	0.1114	0.1013	0.1339	0.1378	0.1106

Supplementary Table 3 FRAP analysis deletion mutants

	WT	ΔPE/ANKbs	ΔABD	PE/ANKbs-only	ΔPS (not in figures)
One-phase association					
Best-fit values					
Y0	-0.05615	-0.01408	-0.1411	-2.811	-0.03120
Plateau	0.7485	0.7320	0.8718	0.8699	0.7179
K	0.01662	0.01220	0.02823	0.0004781	0.01296
Tau	60.18	81.94	35.43	2091	77.13
Half-time (seconds)	41.71	56.80	24.56	1450(msec)	53.47
Span	0.8046	0.7461	1.013	3.681	0.7491
Std. Error					
Y0	0.01209	0.01963	0.02240	0.3488	0.02405
Plateau	0.003295	0.009428	0.002599	0.002656	0.01029
K	0.0003987	0.0006632	0.0007211	2.293e-005	0.0008132
Span	0.01091	0.01635	0.02164	0.3483	0.02037
95% Confidence Intervals					
Y0	-0.07986 to -0.03244	-0.05258 to 0.02442	-0.1850 to -0.09716	-3.495 to -2.126	-0.07838 to 0.01598
Plateau	0.7420 to 0.7549	0.7135 to 0.7505	0.8667 to 0.8769	0.8647 to 0.8751	0.6977 to 0.7380
K	0.01584 to 0.01740	0.01090 to 0.01350	0.02681 to 0.02964	0.0004332 to 0.0005231	0.01137 to 0.01456
Tau	57.48 to 63.15	74.05 to 91.72	33.74 to 37.30	1912 to 2309	68.68 to 87.96
Half-time	39.84 to 43.77	51.33 to 63.57	23.38 to 25.85	1325 to 1600(msec)	47.61 to 60.97
Span	0.7832 to 0.8260	0.7140 to 0.7781	0.9704 to 1.055	2.997 to 4.364	0.7091 to 0.7890
Goodness of Fit					
Degrees of Freedom	2205	1437	1341	1533	1149
R square	0.8218	0.6519	0.8362	0.5420	0.6214
Absolute Sum of Squares	12.62	20.83	6.211	14.52	18.69
Sy.x	0.07565	0.1204	0.06806	0.09732	0.1275

Supplementary Table 4 Reagents and Resources

Antibodies	Source	Identifier
Mouse anti-SPTBN1	BD Bioscience	BD-612563
Rabbit anti-SPTBN1	Abcam	AB-72239
Rabbit anti-SPATN1	Invitrogen	PA5-35383
Rabbit-anti β-Actin	Cell Sign	D6A8
Mouse-anti Tubulin	Sigma	T9026
Mouse Clathrin-heavy chain	Thermo Fisher	clone X22
Phalloidin AlexaFluor488	Invitrogen	A12379
Phalloidin AlexaFluor568	Invitrogen	A12380
Rabbit-anti mouse HRP	BioRad	1706516
Mouse-anti rabbit HRP	BioRad	1706515
Donkey anti-mouse AlexaFluor488	Thermo Fischer	A21202
Donkey anti-rabbit AlexaFluor488	Thermo Fischer	A21206
Donkey anti-mouse AlexaFluor647	Thermo Fischer	A31571
Donkey anti-rabbit AlexaFluor647	Thermo Fischer	A31573
Donkey anti-mouse Cy3	Jackson Imm Res	715-165-150
Donkey anti-rebbit Cy3	Jackson Imm Res	711-165-152

RINGER buffer for Live Microscopy	COMPOSITION
1x	150 mM NaCl, 1mM $MgCl_2$, 1mM $CaCl_2$, 20 mM Hepes (pH 7.4), 5 mM KCl and 2g/l glucose
0.5x	75 mM NaCl, 1mM $MgCl_2$, 1mM $CaCl_2$, 10 mM Hepes (pH 7.4), 2.5 mM KCl and 1g/l glucose
0.1x	15 mM NaCl, 1mM $MgCl_2$, 1mM $CaCl_2$, 2 mM Hepes (pH 7.4), 0.5 mM KCl and 0.2g/l glucose
1.5x	225 mM NaCl, 1mM $MgCl_2$, 1mM $CaCl_2$, 30 mM Hepes (pH 7.4), 7.5 mM KCl and 3g/l glucose

Primers	Sequence 5'>3'
Fwd SPTBN1 FL (pEGFP-C3 and pmCherry-C3)	AAAAAGCTTGCCGCC ATGACGACCACAGTAGCC
Rev SPTBN1 FL	AAACCGCGGTTTCTTTTTGCCAAAAAGGCTGAACGG
Fwd SPTBN1 ΔABD	AAGCTTATGAAGGCCTTAGCTGTTGAAG
Rev SPTBN1 ΔABD	AAAACCGCGGTCATTTCTTTTTGCCAAAAA
Fwd SPTBN1 ΔPE/ANKbs	AACTCTGGACATTCAGATGCCGCCACCCTGGTGGACACAGGGGACAAGTT
Rev SPTBN1 ΔPE/ANKbs	GGTGGCGGCATCTGAATGTCCAGAGTT
Fwd SPTBN1 PE/ANKbs	AAACTCGAGATGATCGCTGAATGGAAGGATGGCCTC
Rev SPTBN1 PE/ANKbs	AAACCTAGGCCGCACCCTGCGGCTCTCACA
Fwd SPTBN1 ΔPS	GAGCTCATAAGACAGGAGAAACTGGAACTGCAGAAGATATTCCAGGAAAT
Rev SPTBN1 ΔPS	TTCCAGTTTCTCCTGTCTTATGAGCTC

Plasmids	Source	Identifier
GFP-SPTBN1	This manuscript	
GFP-Actin	MBI	
RFP-Actin	MBI	
mCherry-SPTBN1	This manuscript	
GFP-PM (Lyn)	Addgene	21213
Scarlet-PM (Lck)	Addgene	98821
GFP-SPTBN1 ΔPE/ANKbs	This manuscript	
GFP-SPTBN1 ΔPS	This manuscript	
GFP-SPTBN1 ΔABD	This manuscript	
GFP-SPTBN1 PE only	This manuscript	
mCherry-AP2σ	DiFiore' Lab	
LifeAct-Ruby	MBI	
RFP-Myosin Light Chain	MBI	

Reagents	Source	Identifier
Nitric acid	Sigma	438073
Latrunculin A	Sigma	L5163
Blebbistatin	Sigma	B0560
Jasplakinolide	Sigma	J4580
Nocodazole	Sigma	M1404
Diamide	Sigma	D3648
Hexamethyldisilazane	Sigma	440191
Sylgard 184 silicone elastomer kit	Dow Corning	1064291
PEG-PLL	Ruixibio	R-PL1226
Silicone membrane	SMI	n.a.
Dow Corning® high-vacuum silicone grease	Sigma	Z273554-1EA
FM4-64 FX	Thermo Scientific	F34653

Cell culture Reagents	Source	Identifier
DMEM High Glucose	Lonza	BE12-614F
HAM'S F12	Biowest (VWR)	L0136-500
Leibovitz L15	Biowest (VWR)	L0300-500
Endothelial Cell Growth Medium	Cell Applications, inc	211-500
Fetal Bovine Serum	Euroclone	ECS0182L
Horse Serum	Life Technologies	16050-122
Cholera Toxin	Sigma-Aldrich	C8052-2MG
Holo Transferrin	Sigma-Aldrich	T0665-100MG
Hydrocortisone	Sigma-Aldrich	H0888-1G
Insulin (Bovine pancreas)	Sigma-Aldrich	I0516-5ML
L-Glutamine	Euroclone	LOBE17605F
Sodium Pyruvate	Microtech	L0642
Trypsin-EDTA	Euroclone	ECB3052D-20
Penicillin Streptomycin	Euroclone	ECB3001L
Puromycin	Vinci-Adipogen	AG-CN2-0078-M100
Fibronectin	Roche	11080938001

Supplementary Table 5 Microscopes, devices and software.

Microscopes	Brand	
TIRF	Leica	
SP5 laser scanning confocal	Leica	
SP8 laser scanning confocal	Leica	
Spinning Disk Confocal Unit	Olympus	
Devices	Source	Patent n˚
Cell compression	IFOM	WO 2019/086702
Cell stretcher	IFOM	WO 2018/149795
Aluminum coverslip holder	Nils Gauthier	
Micropatterning wafer	MBI/IFOM	
Software	Brand	
LAS X	Leica	
CellSense	Olympus	
Prism	GraphPad	
Image Lab (5.0)	Biorad	
Fiji	NIH	
R studio		
Illustrator	Adobe	
