# Supplementary material for: Complementary mesoscale dynamics of spectrin and acto-myosin shape membrane territories during mechanoresponse
Source: Nat Commun. 2020 Oct 9;11:5108. doi: 10.1038/s41467-020-18825-7 (PMC7547731; doi:10.1038/s41467-020-18825-7)
Supplement: Supplementary file 2 — Description of Additional Supplementary Files [file 41467_2020_18825_MOESM2_ESM.pdf]

Description of Additional Supplementary Files

File: Supplementary Movie 1
Description: Fibroblast spreading assay: cell edge analysis of GFP-βII-spectrin and RFP-Actin

File: Supplementary Movie 2
Description: Fibroblast spreading on microprinted fibronectin-coated lines (GFP-βII-spectrin and RFP-Actin)

File: Supplementary Movie 3
Description: Fibroblast spreading: PIV analysis of GFP-βII-spectrin and RFP-Actin flows

File: Supplementary Movie 4
Description: Actin and βII-spectrin dynamics during Latrunculin A and Blebbistatin washout experiments

File: Supplementary Movie 5
Description: Differential βII-spectrin deletion mutants' behavior during spreading

File: Supplementary Movie 6
Description: βII-spectrin-ΔABD displays edge instability during spreading

File: Supplementary Movie 7
Description: Cell compression assay

File: Supplementary Movie 8
Description: Mesoscale dynamics of GFP-βII-spectrin and mCherry-AP2 during osmotic shocks
