# Supplementary material for: Complementary mesoscale dynamics of spectrin and acto-myosin shape membrane territories during mechanoresponse
Source: Nat Commun. 2020 Oct 9;11:5108. doi: 10.1038/s41467-020-18825-7 (PMC7547731; doi:10.1038/s41467-020-18825-7)
Supplement: Supplementary file 12 — Reporting Summary [file 41467_2020_18825_MOESM12_ESM.pdf]

nature research

Corresponding author(s): Nils Gautier, Andrea Ghisleni

Last updated by author(s): 9/9/2020

Reporting Summary

Nature Research wishes to improve the reproducibility of the work that we publish. This form provides structure for consistency and transparency in reporting. For further information on Nature Research policies, see Authors & Referees and the Editorial Policy Checklist.

Statistics

For all statistical analyses, confirm that the following items are present in the figure legend, table legend, main text, or Methods section.

n/a	Confirmed	
☐	☒	The exact sample size (n) for each experimental group/condition, given as a discrete number and unit of measurement
☐	☒	A statement on whether measurements were taken from distinct samples or whether the same sample was measured repeatedly
☐	☒	The statistical test(s) used AND whether they are one- or two-sided<br>*Only common tests should be described solely by name; describe more complex techniques in the Methods section.*
☒	☐	A description of all covariates tested
☒	☐	A description of any assumptions or corrections, such as tests of normality and adjustment for multiple comparisons
☐	☒	A full description of the statistical parameters including central tendency (e.g. means) or other basic estimates (e.g. regression coefficient) AND variation (e.g. standard deviation) or associated estimates of uncertainty (e.g. confidence intervals)
☐	☒	For null hypothesis testing, the test statistic (e.g. F, t, r) with confidence intervals, effect sizes, degrees of freedom and P value noted<br>*Give P values as exact values whenever suitable.*
☒	☐	For Bayesian analysis, information on the choice of priors and Markov chain Monte Carlo settings
☒	☐	For hierarchical and complex designs, identification of the appropriate level for tests and full reporting of outcomes
☐	☒	Estimates of effect sizes (e.g. Cohen's d, Pearson's r), indicating how they were calculated

Our web collection on statistics for biologists contains articles on many of the points above.

Software and code

Policy information about availability of computer code

Data collection	The following softwares were used for data collection:<br>LAS X Leica<br>CellSense Olympus<br>Image Lab (5.0) Biorad<br>Fiji NIH
Data analysis	The following softwares were used for data analysis: Prism Graphpad, Fiji NIH, R studio, Excel Microsoft.<br>Custom- written macros (Fiji and R studio) were used for PIV and radial kymograph analysis.<br>Linear regression were calculated by R studio

For manuscripts utilizing custom algorithms or software that are central to the research but not yet described in published literature, software must be made available to editors/reviewers. We strongly encourage code deposition in a community repository (e.g. GitHub). See the Nature Research guidelines for submitting code & software for further information.

Data

Policy information about availability of data

All manuscripts must include a data availability statement. This statement should provide the following information, where applicable:

- Accession codes, unique identifiers, or web links for publicly available datasets
- A list of figures that have associated raw data
- A description of any restrictions on data availability

The authors declare that data supporting the findings of this study are available within the paper [and its supplementary information files].

All the custom-written software and engineered devices are available from the corresponding author upon reasonable request.

Field-specific reporting

Please select the one below that is the best fit for your research. If you are not sure, read the appropriate sections before making your selection.

☒ Life sciences ☐ Behavioural & social sciences ☐ Ecological, evolutionary & environmental sciences

For a reference copy of the document with all sections, see nature.com/documents/nr-reporting-summary-flat.pdf

Life sciences study design

All studies must disclose on these points even when the disclosure is negative.

Sample size	No statistical methods were used to predetermine sample size.
Data exclusions	For live-microscopy experiments incomplete (due to technical fault) or toxicity hampered experiments were excluded from the analysis.<br>For quantitative analysis, outlier exclusion info are listed in the manuscript (Extended Data Tables or Figure Legends).
Replication	All experiments were done in independent triplicate or more. A single experiment is intended as independently prepared sample.<br>Specifically for live-microscopy experiments, three or more independent transfection of fluorescently-tagged proteins were performed during non-consecutive days. For non-live experiments three or more experimental procedure (i.e. immunostaining or immunoblotting) were performed on independently prepared samples.
Randomization	Not relevant to the study
Blinding	Investigator is blinded during data analysis

Reporting for specific materials, systems and methods

We require information from authors about some types of materials, experimental systems and methods used in many studies. Here, indicate whether each material, system or method listed is relevant to your study. If you are not sure if a list item applies to your research, read the appropriate section before selecting a response.

Materials & experimental systems

n/a	Involved in the study	
☐	☒	Antibodies
☐	☒	Eukaryotic cell lines
☒	☐	Palaeontology
☒	☐	Animals and other organisms
☒	☐	Human research participants
☒	☐	Clinical data

Methods

n/a	Involved in the study	
☒	☐	ChIP-seq
☒	☐	Flow cytometry
☒	☐	MRI-based neuroimaging

Antibodies

Antibodies used	Mouse anti-SPTBN1 BD Bioscience BD-612563<br>Rabbit anti-SPTBN1 Abcam AB-72239<br>Rabbit anti-SPATN1 Invitrogen PA5-35383<br>Rabbit-anti β-Actin Cell Sign D6A8<br>Mouse-anti Tubulin Sigma T9026<br>Mouse Clathrin-heavy chain Thermo Fisher clone X22<br>Phalloidin AlexaFluor488 Invitrogen A12379<br>Phalloidin AlexaFluor568 Invitrogen A12380<br>Rabbit-anti mouse HRP BioRad 1706516<br>Mouse-anti rabbit HRP BioRad 1706515<br>Donkey anti-mouse AlexaFluor488 Thermo Fischer A21202<br>Donkey anti-rabbit AlexaFluor488 Thermo Fischer A21206<br>Donkey anti-mouse AlexaFluor647 Thermo Fischer A31571<br>Donkey anti-rabbit AlexaFluor647 Thermo Fischer A31573<br>Donkey anti-mouse Cy3 Jackson Imm Res 715-165-150<br>Donkey anti-rebbit Cy3 Jackson Imm Res 711-165-152
Validation	We report here all the validation and testing conditions provided by the manufacturer's website<br>BD-612563: western blot (WB) routinely tested, immunofluorescence (IF) tested during development

AB-72239: WB 1:2000-10000 detects band of approximately 274 kDa, IF 1ug/ml
PA5-35383: this antibody was verified by Cell Treatment to ensure that the antibody binds to the antigen stated, IF 1:250, WB 1:5000
D6A8: WB 1:1000, IF 1:200, Monoclonal antibody is produce by immunizing animals with a synthetic peptide corresponding to residues near the N-teminus of human beta-Actin protein
T9026: verified IF 1:500 using chicken fibroblasts, WB 1:500 using human or chicken fibroblasts
Thermo Fisher clone X22: verified IF 1:1000, WB 1:100-500

Eukaryotic cell lines

Policy information about cell lines

Cell line source(s)	Cell lines were derived from IFOM Biobank under the supervision of IFOM Cell Biology Unit. Mouse Embryonic Fibroblast RPTP α +/+ background were obtained from Prof. Mike Sheetz (JCellBiol. 2003 14;161(1):143-53)
Authentication	Authentication was performed by GenePrint 10 System (Promega)
Mycoplasma contamination	I hereby confirm all cell lines used in this manuscript were negatively tested for mycoplasma contamination
Commonly misidentified lines (See ICLAC register)	none of the commonly misidentified lines has been used in the present study
