# Supplementary figures and images for: Complementary mesoscale dynamics of spectrin and acto-myosin shape membrane territories during mechanoresponse
Source: Nat Commun. 2020 Oct 9;11:5108. doi: 10.1038/s41467-020-18825-7 (PMC7547731; doi:10.1038/s41467-020-18825-7)

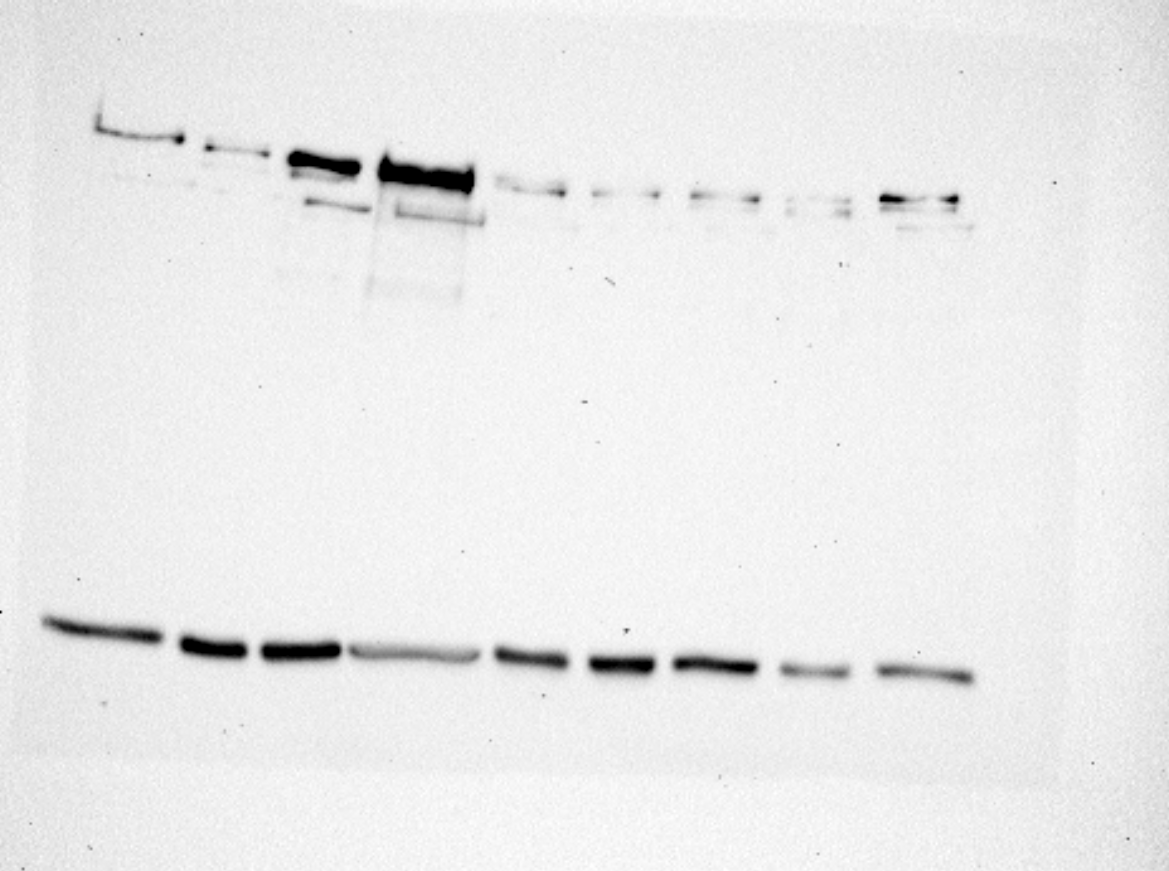

Supplement: Supplementary file 13 — Source Data [file 41467_2020_18825_MOESM13_ESM.zip › Ghisleni et al Source Data/Figure S2/S2B-WB_cell lines sptbn1 + tubulin.tif]
